# Standard Treatment Workflows: scaling system-compatible approaches to rational antibiotic use

**DOI:** 10.3389/fmed.2026.1789243

**Published:** 2026-04-22

**Authors:** Hitesh K. Sharma, Ravinder Singh, Dhiraj Kumar, Daanish, Anjali Bajaj, Mohan Kant, Viney Lather, Virinder S. Parmar, Jerin J. Cherian, Kamini Walia, Ashoo Grover

**Affiliations:** 1 Indian Council of Medical Research, New Delhi, India; 2 Armed Forces Medical College, Pune, India; 3 Department of Health and Family Welfare, Government of Himachal Pradesh, Shimla, India; 4 Amity Institute of Pharmacy, Amity University Uttar Pradesh, Noida, India; 5 Nanoscience Program, CUNY Graduate Center, Department of Chemistry, City College & Medgar Evers College, The City University of New York, New York, NY, United States

**Keywords:** antimicrobial resistance, antimicrobial stewardship, clinical guidance scaling, health systems, Standard Treatment Workflows

## Abstract

Antimicrobial resistance (AMR) threatens the effectiveness of essential medicines, particularly in resource-constrained health systems where high patient volumes, limited diagnostic capacity and inconsistent guideline adherence influence antibiotic prescribing. India faces a substantial AMR burden, with national surveillance reporting carbapenem resistance exceeding 80% in *Klebsiella pneumoniae* and over 90% in *Acinetobacter baumannii* bloodstream isolates. In response, the Indian Council of Medical Research (ICMR) has developed Standard Treatment Workflows (STWs) concise, evidence-based, point-of-care clinical guidance intended to support rational antibiotic use across levels of care. This Practise paper examines the stewardship orientation, system compatibility and readiness for scale-up of STWs to inform their wider adoption. We reviewed 157 STWs addressing conditions requiring antibiotic therapy and mapped recommended agents against the WHO Access-Watch-Reserve (AWaRe) classification, the National List of Essential Medicines (NLEM) and Indian Public Health Standards (IPHS). The recommended antibiotics predominantly fall within the WHO Access category and are largely drawn from medicines listed in India’s NLEM. These features indicate strong alignment of STWs with antimicrobial stewardship principles and existing health system capacities. ICMR Standard Treatment Workflows are a stewardship-aligned, system-compatible clinical guidance tool with strong readiness for scale-up; this paper argues for their adoption in India and their relevance for other high-burden health systems.

## Introduction

Antimicrobial resistance (AMR) is among the most serious global public health challenges of the 21st century. Recent estimates suggest that by 2050 more than 39 million deaths could be directly attributable to drug-resistant infections, with the greatest burden falling on low- and middle-income countries (LMICs) (1–4). South and Southeast Asia already report some of the highest resistance rates globally, including resistance to last-resort antibiotics (5, 6).

India’s national surveillance data highlight the urgency of the situation. In 2024, carbapenem resistance exceeded 80% in *Klebsiella pneumoniae* and 91% in *Acinetobacter baumannii* bloodstream isolates in tertiary hospitals (7, 8). Declining susceptibility of *Escherichia coli* to commonly used antibiotics further narrows effective treatment options.

India has initiated several coordinated national responses to address antimicrobial resistance. The National Action Plan on Antimicrobial Resistance (NAP-AMR) provides the overarching policy framework for strengthening surveillance, stewardship, infection prevention and research across sectors. Complementing this strategy, the ICMR Antimicrobial Resistance Surveillance and Research Network (i-AMRSS) generates nationwide data on resistance patterns from sentinel hospitals, informing national treatment policies and public health planning (7). While these initiatives have significantly strengthened surveillance and policy coordination, translating stewardship principles into consistent prescribing practises at the point of care remains a critical operational challenge. Within this broader national AMR response, Standard Treatment Workflows (STWs) were developed as a practical clinical tool to guide diagnosis and antibiotic selection in routine healthcare settings.

Although AMR is a natural evolutionary phenomenon, its rapid acceleration is largely driven by human behaviour and health system factors, including inappropriate prescribing, empirical treatment without diagnostic support, inconsistent guideline adherence and weak regulatory oversight (9–12). Addressing AMR therefore requires practical, system-level interventions that can shape prescribing behaviour at the point of care, particularly in high-volume, resource-constrained settings.

In this context, the Indian Council of Medical Research (ICMR), under the Ministry of Health and Family Welfare, Government of India, has developed Standard Treatment Workflows (STWs) concise, evidence-based clinical pathways designed to guide diagnosis and treatment for common conditions (13, 14). Rather than evaluating real-world implementation or impact, this Practise paper examines the design quality, stewardship alignment and system compatibility of STWs. ICMR Standard Treatment Workflows are a stewardship-aligned, system-compatible clinical guidance tool with strong readiness for scale-up; this paper argues for their adoption in India and their relevance for other high-burden health systems.

## Understanding drivers of AMR in routine practise

Antimicrobial resistance emerges from a combination of clinical and non-clinical factors operating across healthcare systems (10, 12). Key clinical drivers include empirical prescribing without diagnostic confirmation, unnecessary use of broad-spectrum antibiotics, prolonged treatment durations and inconsistent adherence to treatment guidelines, particularly in high-volume care settings (9–12). Non-clinical drivers such as patient expectations, over-the-counter access to antibiotics, pharmaceutical marketing influences and limited public awareness further contribute to inappropriate antibiotic use (15, 16).

**Figure 1:**
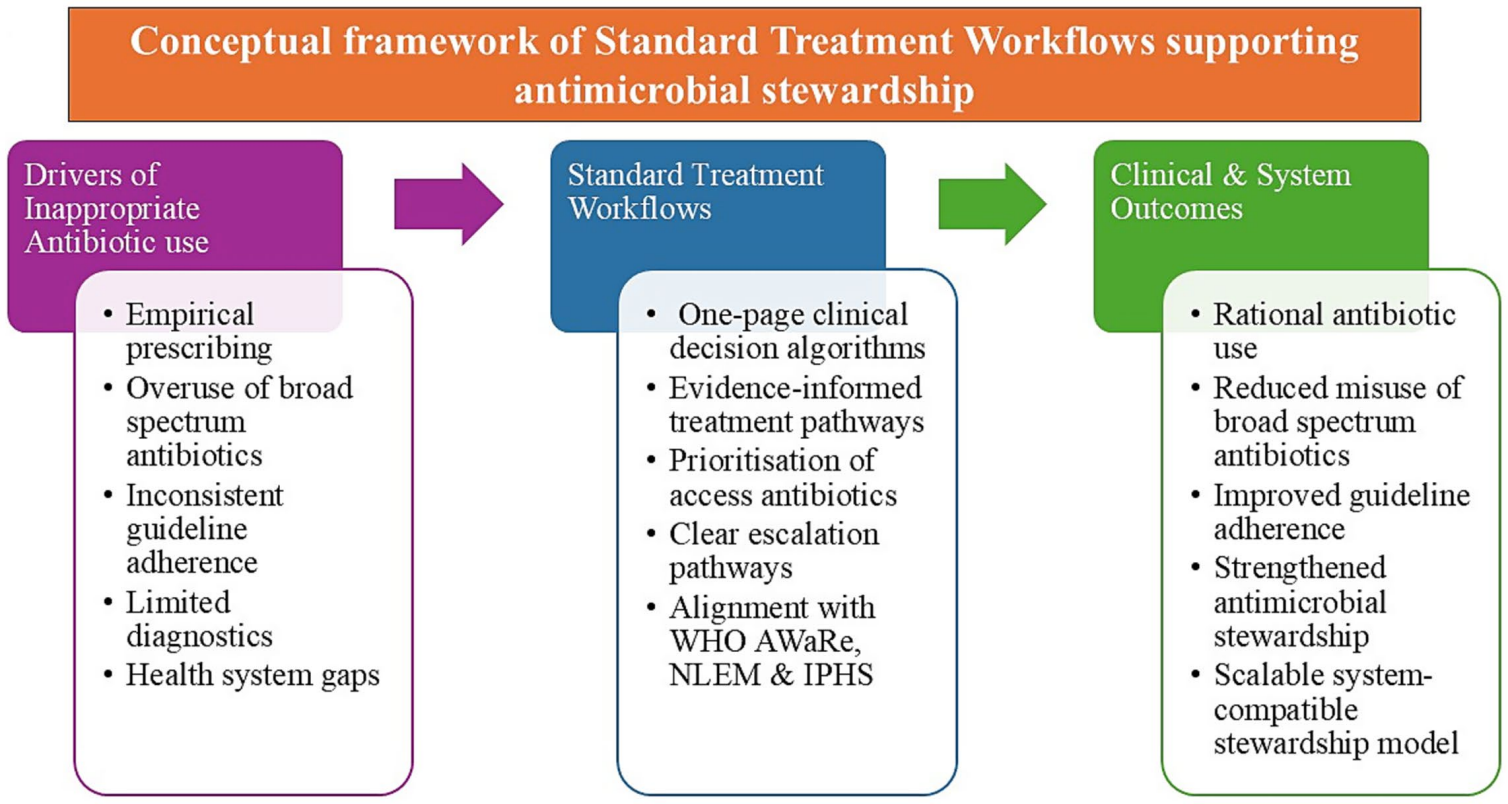
Framework illustrating how Standard Treatment Workflows translate antimicrobial stewardship principles into routine clinical decision-making.

Addressing these drivers requires interventions that influence prescribing behaviour at the point of care. Standard Treatment Workflows were developed with this objective in mind. By translating antimicrobial stewardship principles into concise, condition-based clinical pathways, STWs aim to reduce empirical prescribing, prioritise appropriate first-line therapy and promote more consistent adherence to treatment guidance within routine clinical practise (Figure 1).

## Practise context: Standard Treatment Workflows in India

STWs are one-page, condition-specific clinical algorithms covering common syndromes across primary, secondary and tertiary care. Developed through national expert consultation and periodically updated, they integrate diagnostic cues, first-line treatment choices, referral thresholds and follow-up advice (13, 14). The recommendations within STWs are derived from a synthesis of available clinical evidence, relevant national and international treatment guidelines, and consensus among multidisciplinary expert groups involved in the STW development process. The workflows are periodically reviewed and revised through expert consultation processes coordinated by the Indian Council of Medical Research to incorporate emerging clinical evidence, updated treatment guidelines and evolving antimicrobial resistance patterns.

Each workflow is structured to reflect the diagnostic capacity, medicine availability and referral pathways relevant to primary, secondary and tertiary healthcare facilities, enabling the guidance to be adapted across different levels of the health system.

Unlike traditional clinical guidelines, STWs are designed for rapid use during routine consultations and do not rely on advanced diagnostics or specialist input. Their concise format, visual clarity and alignment with national health system policies are intended to enhance usability and consistency of care across diverse healthcare settings.

## Analysis of antibiotic recommendations in STWs

This paper undertook a descriptive policy and design analysis of the STWs developed by the Indian Council of Medical Research (ICMR). All STWs publicly available at the time of manuscript preparation were retrieved from the official ICMR website.^1^ The analysis included the complete STW compendium covering 157 clinical conditions across multiple specialties.

Workflows that contained antibiotic treatment recommendations were included in the analysis. STWs addressing conditions that did not involve antibiotic therapy were excluded. For each included workflow, antibiotic agents listed within the treatment pathways were manually extracted and compiled into a structured dataset. The analysis included all antibiotics mentioned in the workflows, including both first-line and alternative treatment recommendations. This approach ensured that all antibiotic options recommended within the STWs were captured for classification according to the WHO AWaRe framework and for comparison with the NLEM and IPHS.

Each antibiotic was subsequently classified according to the World Health Organisation Access-Watch-Reserve (AWaRe) framework. In addition, the presence of each antibiotic in the NLEM and its availability across levels of care according to the IPHS were documented. The resulting dataset was used to assess stewardship alignment and implementation feasibility (Supplementary Table S1).

## Stewardship orientation and system compatibility of STWs

### Prioritisation of narrow-spectrum, first-line therapy

Across the workflows analysed, antibiotic recommendations were predominantly drawn from the WHO Access category. In total, 29 distinct antibiotics were identified across the analysed workflows. According to the WHO AWaRe classification, 41% of these antibiotics belonged to the Access category, 55% to the Watch category, and approximately 4% to the Reserve category. In addition, 91% of the recommended antibiotics were included in the National List of Essential Medicines (NLEM), demonstrating strong alignment between STW prescribing guidance and national medicine policy.

STWs consistently prioritise Access-category antibiotics, emphasising narrow-spectrum first-line therapy with clearly defined treatment durations and criteria for escalation to broader-spectrum agents. Although Access-category antibiotics are prioritised, several workflows appropriately recommend Watch-category antibiotics in specific clinical contexts. These include conditions such as severe pneumonia, sepsis, complicated urinary tract infections or situations where resistant pathogens are suspected. In such cases, the use of broader-spectrum agents reflects clinical severity and the need for effective empirical therapy while awaiting diagnostic confirmation. This approach is consistent with the WHO AWaRe framework, which recognises that Watch antibiotics remain essential for treating serious infections but should be used judiciously and within clearly defined clinical indications.

STWs are designed to support, rather than replace, clinical judgement. The workflows typically recommend first-line empirical therapy based on common pathogens and clinical presentation, while also outlining criteria for escalation, referral or modification of treatment when patient condition, diagnostic findings or response to therapy indicate the need for alternative antibiotics.

For example, pharyngitis workflows recommend diagnostic criteria prior to prescribing amoxicillin, reserving macrolides for penicillin-allergic patients (17). Uncomplicated urinary tract infections prioritise nitrofurantoin or trimethoprim-sulfamethoxazole for short, defined courses. Pneumonia and sepsis workflows specify stepwise escalation based on severity and level of care.

### Alignment with health system capacity

A defining feature of STWs is their alignment with the NLEM and IPHS. Because medicines listed in the NLEM are typically prioritised for public procurement and price regulation in India, this alignment also indirectly supports affordability and cost-sensitive prescribing within the public health system ensuring that recommended antibiotics are routinely available at the intended level of care. In this review, 91% of antibiotic recommendations were NLEM-listed. Diagnostic steps within STWs are similarly calibrated to infrastructure available at primary, secondary or tertiary facilities.

This alignment addresses a key barrier to guideline adherence in LMICs, the disconnect between clinical recommendations and medicine availability or service capacity (18).

### Readiness for scale-up and transferability

STWs are intentionally designed for scale. Their one-page format, focus on high-yield clinical features and avoidance of resource-intensive diagnostics support adoption across diverse settings (13, 14). With contextual adaptation to local resistance patterns and procurement systems, the STW model is transferable to other health systems seeking practical, system-compatible approaches to antimicrobial stewardship.

Implementation of Standard Treatment Workflows is supported through national dissemination initiatives led by the Indian Council of Medical Research and the Ministry of Health and Family Welfare. The workflows are intended for use across multiple levels of healthcare delivery, particularly in primary and secondary care settings where clinical decision making often occurs under time constraints and with limited diagnostic support. Their concise one-page format allows rapid consultation during routine clinical practise and makes them suitable for incorporation into training and clinical orientation programmes. However, successful implementation may depend on several factors, including clinician awareness, institutional adoption, availability of recommended medicines and integration into digital clinical decision-support systems. Addressing these operational factors through training, dissemination and digital integration could further strengthen the role of STWs in improving antimicrobial stewardship (19).

## Conclusion

Antimicrobial resistance reflects not only microbial evolution but also how clinical decisions are structured within health systems. While the development of new antibiotics remains essential, improving the use of existing medicines represents an immediate and achievable priority for strengthening antimicrobial stewardship.

By standardising first-line antibiotic choices, aligning recommendations with essential medicine policies and tailoring guidance to realistic service capacities, Standard Treatment Workflows (STWs) are designed to address several operational barriers that have historically limited the effectiveness of stewardship initiatives. Their concise, workflow-based format, combined with strong alignment with the National List of Essential Medicines and Indian Public Health Standards, suggests that STWs can serve as a practical clinical guidance tool to support more consistent antibiotic prescribing across different levels of care.

Although further implementation research will be important to evaluate their impact on prescribing behaviour and clinical outcomes, the design and system compatibility of STWs indicate promising potential to support antimicrobial stewardship in routine clinical practise.

This paper therefore highlights the potential value of integrating STWs into clinical training programmes, digital clinical decision-support systems and health system quality assurance mechanisms. With appropriate contextual adaptation, the STW approach may also offer a useful framework for other health systems seeking practical and potentially scalable strategies to improve rational antibiotic use.

## Footnotes

1
https://www.icmr.gov.in/standard-treatment-workflows-stws
