# Supplementary material for: Standard Treatment Workflows: scaling system-compatible approaches to rational antibiotic use
Source: Front Med (Lausanne). 2026 Apr 22;13:1789243. doi: 10.3389/fmed.2026.1789243 (PMC13143708; doi:10.3389/fmed.2026.1789243)
Supplement: Supplementary file 1 [file Table_1.docx]

**Standard Treatment Workflows: Scaling System-Compatible Approaches to Rational Antibiotic Use**

*Hitesh K Sharma¹, Ravinder Singh¹, Dhiraj Kumar¹, Daanish², Anjali Bajaj³, Mohan Kant¹, Viney Lather⁴, Virinder S Parmar^5^, Jerin J Cherian¹, Kamini Walia¹, Ashoo Grover¹**

*¹Indian Council of Medical Research, New Delhi, India
²Armed Forces Medical College, Pune, India
³Department of Health and Family Welfare, Government of Himachal Pradesh, Shimla, India
⁴Amity Institute of Pharmacy, Amity University Uttar Pradesh, Noida, India*

*^5^Nanoscience Program, CUNY Graduate Center and Department of Chemistry, City College & Medgar Evers College, The City University of New York, 160 Convent Avenue, New York, NY 10031, USA*

****Corresponding Author:*** *Dr. Ashoo Grover*

***Correspondence to:*** *Dr. Ashoo Grover, Scientist G & Head, Delivery Research Division, Indian Council of Medical Research, V. Ramalingaswami Bhawan, P.O. Box No. 4911, Ansari Nagar, New Delhi - 110029, India.*

***Email:*** [*ashoogrover@gmail.com*](mailto:ashoogrover@gmail.com)

**Table S1:** Alignment of Antibiotic Prescribing in ICMR Standard Treatment Workflows with WHO AWaRe, IPHS and NLEM

| **Antibiotics** | **Conditions form Antibiotics are recommended in ICMR STW** | **Indian Public Health Standards** | | | **National List of Essential Medicine** | | | **WHO AWaRe Classification** |
| --- | --- | --- | --- | --- | --- | --- | --- | --- |
|  |  | **Health Care level** | | | **Health Care level** | | |  |
|  |  | **P** | **S** | **T** | **P** | **S** | **T** |  |
| Penicillin G (Benzylpenicillin) | - Pharyngitis and sore throat - Neck Space Infection | ✔ | ✔ | ✔ | ✔ | ✔ | ✔ | Access |
| Ampicillin | - Urinary tract infections - Severe Pneumonia (*children*) - Pyogenic Meningitis - Severe acute malnutrition with complications - Severe Pneumonia (*children*) | - | ✔ | ✔ | ✔ | ✔ | ✔ | Access |
| Amoxicillin | - Acute Rhinosinusitis - Chronic Rhinosinusitis - Acute Otitis Media - Pharyngitis and sore throat - Severe Pneumonia (*children*) - Chronic Obstructive Pulmonary Disease - Respiratory Failure - Acute Respiratory Infections (ARIs) *(Adults)* | ✔ | ✔ | ✔ | ✔ | ✔ | ✔ | Access |
| Cloxacillin | - Sepsis & Septic Shock (*children*) | ✔ | ✔ | ✔ | ✔ | ✔ | ✔ | Access |
| Piperacillin | - ARI *(Adults)* - Pyelonephritis *(At tertiary level for adults only)* | - | - | - | - | - | - | Watch |
| Amoxicillin + Clavulanic acid | - ARI *(Adults)* | ✔ | ✔ | - | ✔ | ✔ | ✔ | Access |
| Ampicillin + Sulbactam | - Acute Rhinosinusitis - Chronic Rhinosinusitis - Urinary tract infections - Sepsis & Septic Shock (*children*) - Severe Pneumonia (*children*) - ARI *(Adults)* | - | - | - | - | - | - | Access |
| Piperacillin + Tazobactam | - ARI *(Adults)* - Respiratory Failure | - | ✔ | - | - | - | ✔ | Watch |
| Cephalexin | - Urinary tract infections | - | - | - | - | - | - | Access |
| Cefuroxime | - Urinary tract infections - Severe Pneumonia (*children*) | - | - | ✔ | ✔ | ✔ | ✔ | Watch |
| Ceftriaxone | - Pharyngitis and sore throat - Urinary tract infections - Pyogenic Meningitis - Acute Diarrhoea (*children*) - Sepsis & Septic Shock (*children*) - Severe acute malnutrition with complications - Severe Pneumonia (*children*) - Respiratory Failure - ARI *(Adults)* | ✔ | ✔ | - | - | ✔ | ✔ |  |
| Cefotaxime | - Pharyngitis and sore throat - Sepsis & Septic Shock (children) - ARI *(Adults)* | ✔ | ✔ | ✔ | ✔ | ✔ | ✔ | Watch |
| Ceftazidime | - ARI *(Adults)* | - | ✔ | ✔ | - | ✔ | ✔ | Watch |
| Cefixime | - Acute Diarrhoea (*children*) | ✔ | ✔ | ✔ | - | ✔ | ✔ | Watch |
| Cefepime | - Respiratory Failure - **Respiratory Infections (RTI)** *(Adults)* | - | - | - | - | - | - | Watch |
| Meropenem | - ARI *(Adults)* | - | - | - | - | - | ✔ | Reserve |
| Erythromycin | - Otorrhoea - Acute Diarrhoea (children) - Severe Pneumonia (children) - ARI *(Adults)* | - | - | - | ✔ | ✔ | ✔ | Watch |
| Clarithromycin | - ARI *(Adults)* | - | ✔ | ✔ | - | ✔ | ✔ | Watch |
| Azithromycin | - Acute Rhinosinusitis (*opted for patients intolerant/ sensitive to penicillin)* - Pharyngitis and sore throat (*opted for patients intolerant/ sensitive to penicillin)* - Severe Pneumonia (children) - Chronic Obstructive Pulmonary Disease - ARI *(Adults)* | ✔ | ✔ | ✔ | ✔ | ✔ | ✔ | Watch |
| Roxithromycin | - Chronic Rhinosinusitis | - | - | - | - | - | - | Watch |
| Ciprofloxacin | - Otorrhoea - Urinary tract infections - Acute Diarrhoea (*children*) | ✔ | ✔ | ✔ | ✔ | ✔ | ✔ | Watch |
| Levofloxacin | - Acute Rhinosinusitis (*opted for patients intolerant/ sensitive to penicillin)* - Urinary tract infections - Respiratory Failure - ARI *(Adults)* | - | ✔ | ✔ | ✔ | ✔ | ✔ | Watch |
| Moxifloxacin | - Respiratory Failure - ARI *(Adults)* | - | ✔ | ✔ | ✔ | ✔ | ✔ | Watch |
| Gentamicin | - Neck Space Infection - Urinary tract infections - Sepsis & Septic Shock (*children*) - Severe acute malnutrition with complications - Severe Pneumonia (*children*) | ✔ | - | - | ✔ | ✔ | ✔ | Access |
| Amikacin | - ARI *(Adults)* | - | ✔ | ✔ | - | ✔ | ✔ | Access |
| Tetracycline | - Acute Diarrhoea (*children*) | - | ✔ | ✔ | - | ✔ | ✔ | Access |
| Doxycycline | - Acute Diarrhoea (*children*) - Chronic Obstructive Pulmonary Disease - ARI *(Adults)* | ✔ | ✔ | ✔ | ✔ | ✔ | ✔ |  |
| Vancomycin | - Pyogenic Meningitis | - | ✔ | - | - | ✔ | ✔ | Watch |
| Nitrofurantoin | - Urinary tract infections | - | ✔ | ✔ | ✔ | ✔ | ✔ | Access |
| Metronidazole | - Chronic Rhinosinusitis *(If maxillary sinusitis is linked to a first-molar dental infection, add metronidazole for anaerobic coverage)* - Neck Space Infection - Acute Diarrhoea (*children*) - Severe acute malnutrition with complications | ✔ | ✔ | ✔ | ✔ | ✔ | ✔ | Access |
| ******* ***P = Primary level of care; S = Secondary level of care; T = Tertiary level of care Defined according to Indian Public Health Standards (IPHS).*** | | | | | | | | |
